# Development and feasibility of Inlife: A pilot study of an online social support intervention for informal caregivers of people with dementia

**DOI:** 10.1371/journal.pone.0183386

**Published:** 2017-09-08

**Authors:** Alieske E. H. Dam, Martin P. J. van Boxtel, Nico Rozendaal, Frans R. J. Verhey, Marjolein E. de Vugt

**Affiliations:** Department of Psychiatry and Neuropsychology/Alzheimer Centre Limburg, School for Mental Health and Neuroscience, Maastricht University, Maastricht, The Netherlands; University of Stirling, UNITED KINGDOM

## Abstract

**Background:**

Informal caregivers of individuals with dementia have an increased risk to face social isolation due to progression of the disease. Online social media interventions might offer a new opportunity to increase access to social support and enhance positive interactions and openness in dementia care networks.

**Objective:**

This explorative pilot study describes (1) the development of an online social support intervention Inlife, and (2) the evaluation of the feasibility of this intervention and the measurements to assess its effectiveness.

**Methods:**

The Medical Research Council (MRC) framework guided the development of the online social support intervention. This is a stepwise approach that integrates potential users’ views with the development and validation of the program content. The program was developed by combining (1) individual caregiver interviews (n = 10), (2) focus group sessions with experts and web designers (n = 6), and (3) individual think-aloud tests (n = 2). Subsequently, a pilot study with informal caregivers was conducted (n = 25) to examine the program’s feasibility and preliminary effectiveness. Online self-report measures were completed at baseline and at four follow-up time points.

**Results:**

In total, 23 participants completed the newly developed Inlife intervention. Despite the high number of low-active users (17/23, 73%), Inlife had a good feasibility score of 7.1 (range: 1–10). The Calendar and Timeline were used most frequently and contributed to better care coordination and positive interactions.

**Conclusions:**

Although the Inlife platform received a sufficient feasibility rating, the uptake was not optimal. Therefore, the Inlife platform was adapted to limit the number of low-active users and improve user friendliness. Recommendations for additional treatment adherence were provided. The development according to the MRC framework and the sufficient feasibility rating of Inlife formed the basis for a future effectiveness study.

## 1. Introduction

Family members and other social network members are increasingly involved in the care of individuals with dementia. Informal care has gained significant public health value due to the rising number of people with dementia and the decreasing availability of formal carers in the aging population [1]. Although an accumulative body of literature demonstrates that informal caring for an individual with dementia can be a rewarding experience leading to enrichment and growth [2, 3], informal caregiving might simultaneously have a negative impact on individuals’ quality of life and well-being. Caregiving can lead to social isolation, burden, depression and poor physical health [4, 5]. Over the caregiving trajectory, interpersonal relationships change, and feelings of loneliness might contribute to depression [5–7]. In contrast, higher levels of subjective social support contribute to better psychological well-being and caregiver health [8]. Consistently, social support theories [9, 10] underline the buffering effects of social support. According to the stress and coping social support theory, social support protects people indirectly from the negative health effects of stressful events (e.g., long-term caregiving) by promoting adaptive coping and appraisals of stressful situations [9–11]. More specifically, general social support research has demonstrated that perceived support is more strongly associated with well-being than actual received support [12]. In particular, perceived quality of relationships and satisfaction with social interactions rather than the quantitative structural characteristics (i.e. network size) contribute to beneficial health outcomes [13]. This highlights the importance of recognizing subjective social support needs to improve caregiver well-being. However, caregivers often face barriers to seek support such as negative past experiences, stigma, fear of burdening others or lack of openness [14, 15]. These barriers might be reduced by facilitating caregivers’ acknowledgement of their support needs and enabling a positive shift of focus towards opportunities, rather than loss [16]. Therefore, there is a need for interventions that focus on improvement of positive interactions and access to social support for caregivers of individuals with dementia.

Advancing online technologies such as social media platforms are promising to improve social support for caregivers of individuals with dementia. Recently, it was demonstrated that online caregiver interventions effectively improve self-esteem, self-efficacy, and feelings of depression [17]. Evidence shows that online support interventions might provide several advantages compared to face-to-face support such as ease of accessibility, regardless of time, physical constraints, or stigma related to (professional) help-seeking [17–19]. Therefore, online interventions might offer new opportunities to strengthen social support and prevent feelings of loneliness in informal carers of individuals with dementia.

In this pilot study, we evaluate the feasibility of a newly developed online social support intervention, ‘Inlife’. Inlife is a web-based platform that promotes social support, positive interactions, and access to information within the dementia caregiver social network. In the present study, we describe the development and the piloting process of the Inlife platform. We closely followed the updated iterative Medical Research Council (MRC) framework for the development and the evaluation of complex interventions [20]. The elements of the MRC framework concerning identification of theory are described elsewhere[15, 21]. The current paper focuses on the development and piloting of the Inlife intervention. These particular elements of the MRC framework are described in next sections of this paper (i.e., implemented in a two-year period August 2014-June 2016, Fig 1). As suggested in a recent literature review on development of technological interventions, we incorporated user views into the development process [22]. Following the MRC framework, this exploratory pilot study forms the basis for a future process and effect evaluation in a randomised controlled trial.

**Fig 1:**
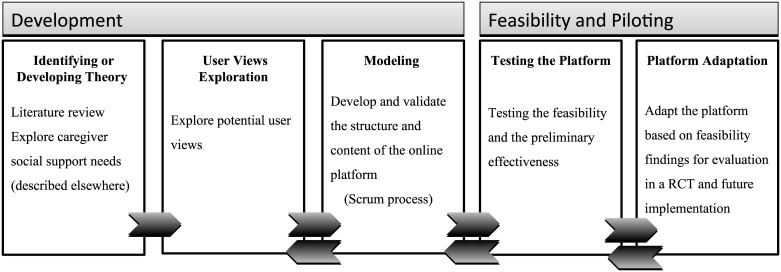
Iterative development and piloting process informed by the elements of the MRC framework.

## 2. Methods and results

The development of Inlife was an iterative process that involved co-creation with potential users. Therefore, the elements of the MRC framework formed a negative feedback loop (Fig 1), as potential users were involved in several phases of the development process, content determination, and validation of the Inlife intervention. The method and results of the elements in the MRC framework are described separately (Fig 1).

### Development

#### Explore potential user views

**Methods:** We collected information on caregiver users’ views regarding Internet and online interventions in semi-structured interviews. For details about the methods and demographics, see [15]. The interviews were transcribed verbatim and analyzed using inductive content analysis by two researchers. Users’ views were also collected during the modeling and piloting phase.

**Results:** In total, eight of the ten interviewed spouses [15] stated that they used the Internet for several reasons such as checking e-mail, searching for information, and playing games. The spouses believed that Internet interventions might be a good addition for sharing information and maintaining contact with others. Internet devices were positively valued due to their accessibility and opportunities to support care:

*“If I would want it*, *I would use the Internet such as Skype or maybe other websites*. *I see the opportunities of modern tools*. *I don’t need it at this moment*. *But I know there are supporting tools (e.g., that prevent falling out of bed)*. *This and other new tools I would try*. *I would have to experience it*.

(spouse, female, 63 years old)

*“Interviewer: Imagine there was a website where you and other involved caregivers such as family members and friends could arrange for help.” “Spouse: I would be in favor of such a system if people in my environment know about the existence of such a system because from both sides it happens in an anonymous setting. For example, you can ask for help in general, and if someone is not available or is not willing, then they don’t feel obligated to respond*.

(spouse, male, 66 years old)

However, caregivers also mentioned concerns regarding unfamiliarity with computer usage, privacy and security:

*“However, on such a website, I would approach people structurally in three phases. First, people in my inner circles and then, somewhat broader, the people involved in my outer circles. Imagine you would develop an app for a smartphone*. *You should think about it carefully that information would not become public”*

*(spouse, male, 75 years old)*.

To some extent, results indicated that spouses were open to online interventions. Accessibility and security of the online intervention should be given high priority in the development process.

#### Modeling the structure and content of the intervention

**Methods:** The development of Inlife was structured according to the Scrum method [23], which is an innovative method to design and evaluate a temporary product in so-called Sprints, short timeframes of four weeks in duration. In multi-disciplinary focus groups consisting of two researchers, two clinicians and two web designers, Inlife was developed in five successive iterative Sprints. Each Sprint commenced with a start session, which was followed by a demo session after four weeks.

During each start session, users’ stories were defined. These were short vignettes that described which caregiver needs had to be met by the Inlife intervention:

“As a potential user, I want to share personal messages in such a way that everybody stays involved.”

“As a potential user, I want to be able to decide to whom I send my request for support.”

Themes in the users’ stories were proposed by the members of the Scrum team based on their clinical experience, a previous literature review [21], interviews conducted during the needs assessment phase [15] and the aforementioned theoretical frameworks [9–11]. The users’ stories were used to set up a description of functionalities on the Inlife platform (Table 1). To validate the content, the multidisciplinary Scrum team provided feedback in a demo session following each Sprint. Spousal caregivers (n = 2), who were recruited from the memory clinic at the Maastricht University Medical Center (MUMC+), provided feedback on the program content in unstructured ‘think-aloud’ sessions [24]. The caregivers evaluated the webpages and functionalities of the online social support platform (www.myinlife.nl) and were free to give their opinion while using the platform. The researcher took field notes to document the reported key points.

**Table 1:** Executed sprints during the iterative development process.

Sprints	Content of focus groups
Sprint 0 (preparation)	Defining product statements of the general aim of the platform
	Define user-stories of the needs and goals of the potential users
	Design the concept
	Plan the consecutive sprints
Sprint 1 (central users)	Design the account
	Develop the personal profile
	Generally manage and host
Sprint 2 (networks and privileges)	Send an invitation to network members in circles
	Create the network account and profile
	Manage circles and privileges
Sprint 3 (valuable interaction)	Structure personal messages
	Design the timeline
Sprint 4 (involvement and support)	Create the infrastructure for notifications
	Design the calendar
	Develop the overview of actions and support
	Arrange the help function

**Results:** The five Sprints resulted in Inlife, an Internet-based social support tool with eight integrated functionalities (Table 2). Inlife aims to lower the threshold to seek support, prevent feelings of loneliness, improve social support, caregiver competence, and access to information. The content is focused on developing positive social interactions and promoting the involvement of the personal care networks in daily life and care activities. The primary caregiver of the individual with dementia is assigned to coordinate the Inlife platform and may decide which network members are invited and what information can be shared.

**Table 2:** Functionalities and content of the Inlife web application.

Functionality	Content
Circles	The coordinating primary caregiver on the Inlife platform can invite other friends, family members, and significant others into their personal network circles. They have the opportunity to assign the invited network members into three ‘circles’ (inner, middle, outer circle) to ensure a distinct level of privacy and privileges (i.e., only the inner circle has access to the Care Book and when posting a message one can decide with which circle(s) the messages is shared).
Profile	On this page, all network members can upload their photograph, personal contact information, relationship details, and wishes and preferences. The person with dementia can refer to the profile pictures as a ‘face board’ to view and recognize their network members.
Timeline	Network members can share photographs and messages about their daily life or past events with the preferred circle(s) to increase their positive interactions and involvement. The person with dementia can view the pictures in a presentation modus that might assist the interaction about activities that have occurred.
Notifications	Personal messages can be shared with either single or multiple individuals. Recipients are notified quickly by e-mail.
Helping	This function provides an overview of the capacity of the Inlife network to offer assistance and support in different areas of interest. The caregiver can indicate in which particular tasks support is required using several categories (household, leisure events, respite, transport, or other). Subsequently, network members can offer their support in (some of) these categories.
Calendar	The Calendar allows for the creation of a shared schedule to plan events and general appointments. In addition, the primary caregiver can post a request for help in a particular category. People who indicate that they are willing to help in that particular category receive an e-mail and may respond.
Care Book	The Care Book provides an overview of all the contact and practical information that is relevant to the care process. It enables the temporary transfer of care tasks to other network members. This function is only accessible among the inner circle.
Compass	The Compass is a concise collection of links to relevant information resources such as existing websites, articles or videos that offer information on several topics related to dementia and caregiving.

In the individual ‘think-aloud’ sessions, the caregivers positively evaluated the functionalities of the program. They reported that they valued the clear layout with simple icons and the uniform color composition of the webpages. Furthermore, they appreciated that the website was secured by the requirement of a personal password, and that their privacy was guaranteed by the usage of separate network circles. However, they expressed concerns about user guidance and the potential threshold to invite other network members directly by e-mail. Therefore, we provided information regarding each functionality at the top of each page. Furthermore, we designed paper postcards that could be used to invite other network members.

### Feasibility and piloting preliminary effectiveness

#### Testing the feasibility of the platform

**Methods:** An uncontrolled pilot study with a repeated measures design was conducted to examine the feasibility of Inlife. The ethical committee of the psychology faculty of Maastricht University approved this study (No: ECP-157 22 03 2015 Al, Dutch trial registration number: NTR5526).

*Participants and procedure*. Caregivers were recruited via an online advertisement, flyers, and e-mails distributed by Maastricht University and local and national Alzheimer societies and community service organizations. We aimed to include 20 participants in the pilot study [25, 26]. They were not involved in the former modeling phase of the Inlife development process. In total, 25 of the 46 (54%) approached caregivers were willing to participate. The characteristics of the participants are listed in Table 3. Inclusion criteria were: (1) being a primary caregiver of an individual with dementia living in the community, and (2) having access to the Internet. Exclusion criteria were: (1) having insufficient knowledge of the Internet and computers, (2) being overburdened (e.g., having a schedule that is too busy to complete measurements), (3) having severe health problems (e.g., surgery or other severe complaints as assessed by the researcher), (4) having fewer than two available persons in the caregiver social network, (5) being unavailable for a period of longer than four weeks, and (6) facing a great likelihood that the individual with dementia would be transferred to a nursing home in the near future. There were no restrictions in terms of type of dementia and caregiver relationship. Feasibility of the Inlife platform was measured with an online self-report questionnaire that was completed after 16 weeks by the primary caregiver. Furthermore, to assess the feasibility of the effect measurements, online self-report questionnaires were collected at baseline as well as 4-week, 8-week, 12-week and 16-week follow-ups. Participants completed these questionnaires within two weeks and automatically received a reminder e-mail after one week. Following the 16-week study period, participants were free to continue their usage of the platform.

**Table 3:** Background characteristics of caregivers (N = 25) and care recipients (N = 24).

Characteristics		N (%) or mean		
		Total (N = 25)^b^	*Low-active group* *(N = 17)*	*High-active group* *(N = 6)*
Caregiver age, years (N = 25)		55.9 (13.9)	54.4 (16.3)	60.2 (7.0)
**Caregiver gender**	Male	13 (52.0)	7 (41.2)	5 (83.3)
	Female	12 (48.0)	10 (58.8)	1 (16.7)
**Caregiver education**	High school	1 (4.0)	-	-
	Lower vocational school	3 (12.0)	3 (17,6)	-
	College	13 (52.0)	8 (47.1)	4 (66.7)
	Graduate school	8 (32.0)	6 (35.3)	2 (33.3)
**Relationship to the care recipient**	Spouse	8 (32.0)	5 (29.4)	2 (33.3)
	Daughter	10 (40.0)	9 (52.9)	1 (16.7)
	Son	6 (24.0)	2 (11.8)	3 (50.0)
	Granddaughter	1 (4.0)	1 (5.9)	-
**Living with the care recipient**	Yes	9 (36.0)	6 (35.3)	2 (33.3)
	No	16 (64.0)	11 (64.7)	4 (66.7)
Hours of care per week		24.7 (37.9)	21.5 (33.3)	17 (23.1)
Significant others in Inlife circles		4.4 (4.4)	2.7 (2.1)^a^	9.00 (5.9)^a^
Care recipient age, years (N = 24)		79.0 (11.6)	79.4 (10.0)	77.7 (17.2)
**Care recipient gender**	Male	6 (25.0)	4 (23.5)	1 (16.7)
	Female	18 (75.0)	13 (76.5)	5 (83.3)
**Care recipient diagnosis**	Alzheimer’s disease (AD)	18 (75.0)	11 (64)	5 (83.3)
	Vascular dementia	1 (4.2)	1 (6)	-
	FTD	1 (4.2)	-	1 (16.7)
	Lewy body dementia	2 (8.3)	2 (12)	-
	Mixed dementia	2 (8.3)	2(12)	-
	Other		1 (6)	-
Care recipient years of diagnosis		2.1 (1.6)	2.4 (1.7)^a^	1.3 (0.8)^a^

^a^ P<0.05 low-active group compared to high-active group

^b^ of the total sample (N = 25), two participants dropped-out of the study. One spousal caregiver was replaced by her own daughter.

*Measures*. Feasibility was evaluated through the Program Participation Questionnaire (PPQ), which was distributed online and adapted for this study. The PPQ was developed in a previous study [25, 27] based on scales that measured perceived usefulness, user friendliness, and acceptance of information technology [27, 28]. The adapted PPQ contained 34 items relating to usability, user friendliness, and satisfaction with the Inlife platform measured on a 5-point Likert scale ranging from 1 (strongly disagree) to 5 (strongly agree). Mean scores (range: 1–5) on the individual PPQ items were calculated using descriptive statistics (Table 4). These mean scores (range: 1–5) were used to identify positive and negative aspects of the program. Mean item scores of 3.5 or higher were considered to be positive, whereas mean items scores of 2.5 or lower were considered to be negative components of the program. Since there is no gold-standard method to evaluate the overall feasibility of e-health interventions, we asked participants to grade the Inlife program on a 10-point scale. A mean score above 6 was considered to be an acceptable level of feasibility. This approach was also adopted in a previous study [29]. Additionally, participants freely provided comments and offered potential improvements. The most frequently given comments are shown in Table 5. After 16 weeks, the burden experienced by filling-out the online self-report questionnaires was measured using a 2-item survey with a 5-point Likert scale ranging from 1 (strongly disagree) to 5 (strongly agree). Furthermore, log data regarding the actual usage of the platform (log ins, page visits and new posts) were collected.

**Table 4:** Inlife program questionnaire.

1 (strongly disagree) 5 (strongly agree)	Low-active group			High-active group		
	N	Mean or (valid %)	SD	N	Mean or (valid %)	SD
I found Inlife useful/helpful	17	2.65	1.00	6	4.17	0.75
The usage of Inlife made asking for help easier	17	2.06	0.90	6	3.67	1.03
The usage of Inlife made organising help easier	17	2.06	0.90	6	3.67	1.21
Inlife increases involvement of the own social network	17	1.94	0.97	6	4.00	0.63
I have the impression that other people in my network found Inlife useful	17	1.71	0.77	6	4.00	0.63
I used information, advice or tips that were offered by others in the Inlife network	17	1.88	0.99	6	3.50	1.38
I used the circles	17	Yes (52.9)		6	Yes (100)	
		No (47.1)			No (-)	
I found the circles meaningful	15	2.80	1.37	6	3.67	1.03
I filled out my profile	16	Yes (81.2)		6	Yes (100)	
		No (18.8)			No (-)	
I found my Profile meaningful	14	3.07	1.21	6	2.67	1.37
I filled out the Timeline	17	Yes (52.9)		6	Yes (100)	
		No (47.1)			No (-)	
I looked at the Timeline	15	Yes (66.7)		5	Yes (100)	
		No (33.3)			No (-)	
		3.07			3.83	
I found the Timeline meaningful	14		1.39	6		1.33
I looked at the presentation modus of the Timeline	16	Yes (43.8)		6	Yes (83.3)	
		No (56.3)			No (16.7)	
I found the presentation modus of the pictures meaningful	13	2.62	1.19	6	3.17	1.60
I used the Notifications	17	Yes (58.8)		6	Yes (100)	
		No (41.2)			No (-)	
I found the Notifications meaningful	14	3.00	1.30	6	4.33	1.21
I used or looked at the Helping function	17	Yes (64.7)		6	Yes (83.3)	
		No (35.3)			No (16.7)	
I found the Helping function meaningful	13	3.23	1.17	6	2.17	1.60
I looked at the Calendar	17	Yes (94.1)		6	Yes (100)	
		No (5.9)			No (-)	
I used the Calendar to ask for support	16	Yes (18.8)		5	Yes (40.0)	
		No (81.3)			No (60.0)	
I found the Calendar meaningful	14	3.14	1.23	6	4.67	0.82
I looked at the Compass	16	Yes (25.0)		6	Yes (83.3)	
		No (75.0)			No (16.7)	
I found the Compass meaningful	10	2.70	1.34	6	3.17	1.17
I found the goal and the functions of Inlife clear	16	3.38	1.41	6	3.67	1.03
The functions of Inlife do what I had expected	16	2.75	1.39	6	3.50	1.05
How many hours per week did you spend on Inlife?	5	0.52	0.55	4	6.30	11.6
I spend enough time on Inlife to understand the possibilities that Inlife offers	17	3.12	1.41	6	4.17	0.98
The overview in Helping supported me to ask for help more easily	16	2.31	1.08	5	2.60	1.34
The ‘questions for support’ which could be asked in the Calendar helped me to organise care	16	2.06	1.00	6	2.50	1.38
I found the reminder e-mails a good addition	17	3.24	1.35	6	3.17	1.33
I found the bi-weekly update e-mails a good addition	16	3.06	1.34	5	2.80	1.64
I found working with Inlife was easy	17	3.29	1.21	6	4.50	0.55
The start page on Inlife was clear	17	3.35	1.12	6	4.50	0.55
The symbols/icons on Inlife were clear	17	3.41	1.12	6	4.33	0.82
The texts on Inlife were easily readable	17	3.41	1.12	6	4.50	0.55
In general, the context of the texts on Inlife were appealing to me	17	3.18	1.13	6	4.33	0.82
The instructions for Inlife usage were clear to me	17	3.47	1.13	6	4.50	0.55
I found the information that was offered sufficient	17	3.24	1.15	6	4.17	0.75
I have enough technical skills to use Inlife	17	3.59	1.42	6	3.67	1.63
I did not experience problems with privacy on Inlife	17	4.06	1.30	6	4.33	1.21
I experienced no problems with privacy on Inlife during contact with network members	15	4.13	1.25	6	4.00	1.55
I experienced no problems with privacy on the Timeline	16	4.25	1.24	6	4.00	1.55
In general, I am satisfied with the possibilities that Inlife offered	17	2.71	0.92	6	4.33	0.52
Inlife was useful for me	17	1.76	0.83	6	4.33	0.52
I would recommend Inlife to other caregivers of people with dementia	16	3.31	1.25	6	4.50	0.55
How would you grade Inlife on a scale from 1 to 10?	12	6.67	1.30	5	8.00	0.71

**Table 5:** Overview of positive, negative and neutral evaluations of the features of the Inlife platform.

Feature	Positive	Negative	Quotes
**General Satisfaction**	More positive interactions	Low-user activity	*‘Inlife stimulates positive involvement due to sharing of pictures and positive messages*.*’*
	People become more involved (at a distance)	Non-response network members (no habit)	*‘My brothers and sisters get more involved in care and everything else*.*’*
	Better communication with family members	No elaborate user instructions, no videos	*‘Inlife is used limitedly to ask for help*, *but more to share information*.*’*
	Increased sharing of daily experiences	Information transfer is too slow	
		Log in requested too often	
		Threshold/it requires time to build up the platform	
**Circles**	Improved privacy	Not all people accept the invitation	*‘Inlife does not work when not all significant caregivers join*, *then good communication becomes limited*.*’ ‘There were too many steps (log-in) compared to other tools like WhatsApp*.*’*
	Ability to involve a broader network	Difficult to motivate others to join	
		The third circle was not always used	
**Profile**	Easy to adapt	Not everybody adds a profile picture	*‘It is convenient to add pictures to your personal profile*. *The pictures enable the person with dementia to view the persons that join the Inlife circles’*
		Not possible to add e-mail preferences	
		Coordinator and individual with dementia should not be depicted as a dyad	
**Timeline**	More positive sharing due to pictures	Not everybody reads messages, non-response	*‘We use the timeline share recent visits and photo with each other and ‘the individual with dementia can look back at recent activities that have occurred’*
**Helping**	Clear overview of needs and offers	Not all people complete helping	*‘Everybody can see which help is needed*. *This made it more easier to ask for support*, *because you can ask for help to everybody at once*, *and don’t have to ask every single person separately’*
		Sometimes people in circles feel obligated	
**Calendar**	Very useful, especially in the second circle	Not used to request help	*‘The calendar is useful to coordinate by who and when the individual with dementia is visited*.*’*
	Better coordination around care	Not possible to add repeating appointments	
	Convenient and accessible for everybody		
**Notifications**	Useful for personal communication	The title of this function is unclear	*‘It is great that you can decide yourself what is shared with whom*. *Via notifications*, *you can send a message to one person and it’s not necessary to inform the whole circle*.*’*
**Care Book**	Clear overview of contact details	No room to add documents	*‘Easy to report and transfer important contact information (GP*, *daycare) with each other*.*’*
		Not able to share with the third circle	
**Compass**	Nice guideline to find information	Not used by all circle members	*‘ The compass was useful to navigate through available information regarding dementia’*
		More information regarding nursing home placement	
**E-mail reminder**	Necessary to remain up-to-date on new items on Inlife	2 weekly e-mails are redundant	*‘It is easy to receive a notification when a new message is posted’*
		Too many e-mails	

**Results:** In total, 25 caregivers participated, of whom two dropped out of the study. Their reasons for dropout were being too busy (n = 1) and persistent technical problems with the Inlife platform (n = 1). The daughter of this person who dropped out served as a substitute. We could distinguish between ‘active’ (n = 6) and ‘non-active’ (n = 17) Inlife networks. Being active was defined as continued posting of new items on the interactive components of the website (e.g., timeline, notifications, calendar) after a period of eight weeks through the end of the 16-week study period. This cut-off was based on log data from the page visits showing that Inlife usage stabilized after approximately eight weeks. Moreover, two months appeared to be a reasonable timeframe to invite network members, build up and become acquainted with Inlife. Characteristics of the active users and non-active users are listed in Table 3. The most frequently mentioned reasons for being non-active were: (1) non-response of people in the Inlife circles, (2) small number of people in the circles on the Inlife platform (M = 2.7, SD = 2.1), (3) refusal of close significant network members to join or to respond, and (4) usage of other online communication tools (e.g., WhatsApp). In contrast, active users were shown to have significantly larger network circles (M = 9.0, SD = 5.9) and reported clear advantages of the platform, including the (1) clear overview of information in one secure place, (2) more positive involvement, (3) ability to manage and share care from a distance, (4) greater awareness of the situation by others, and (5) less redundant messaging (Table 5). The participants (n = 23) completed 91.3% of the four follow-up measurements. However, 56.5% of these participants received one or more reminder phone-calls, as they did not respond after receiving the reminder e-mail. The survey-questionnaire showed that the online questionnaires to assess effectiveness were moderately burdening (M = 2.8, SD = 0.9) and not difficult (M = 2.3, SD = 1.3) on a scale ranging from 0 (strongly disagree) to 5 (strongly agree). Some users specified that there were too many follow-up measurements, which interfered with the usage of Inlife itself.

The overall participant mean evaluation of the Inlife platform was 7.1 (SD = 1.3) on a 10-point scale, indicating acceptable feasibility. Mean scores on the items used to examine positive and negative aspects of the program are shown in Table 4. Active users evaluated all functionalities more positively than non-active users. However, based on the scores below 2.5, the high-active group indicated that the help function was not viewed as being particularly useful (M = 2.2, SD = 1.6). Similarly, the function to directly request support in the calendar was not evaluated positively in both groups (M = 2.1, SD = 1.0 vs. M = 2.5, SD = 1.4). Table 4 shows that the calendar and timeline function were evaluated the most positively in both groups.

#### Testing the preliminary effectiveness of the platform

**Methods:** To examine the preliminary effectiveness of Inlife, online self-report questionnaires were completed at baseline and at four follow-up time points. Several primary outcome measures were completed. The Multidimensional Scale of Perceived Social Support (MSPSS) (Blumenthal et al., 1987) was used to measure perceived support on a 7-point Likert scale ranging from 1 (very strongly disagree) to 7 (very strongly agree). The scale yields three subscales for family, friends and significant others [30]. Higher scores indicate higher levels of perceived social support. The 12-item Social Support List (SSL-12) was employed to measure received social support, with scores ranging from 1 (seldom or never) to 4 (very often) [31]. The SSL-12 consists of three subscales: everyday support, support in problem situations and esteem support (e.g., receiving compliments), with higher scores reflecting a higher level of received support. The Loneliness Scale (LS) assessed feelings of loneliness on a 5-point Likert scale ranging from 1 (strongly agree) to 5 (strongly disagree) and including an emotional subscale and a social subscale, with higher scores indicating more feelings of loneliness [32]. The 7-item Short Sense of Competence Questionnaire (SSCQ) was used to measure feelings of being capable of caring on three domains: (1) satisfaction with the individual with dementia as a recipient of care, (2) satisfaction with one’s own performance as a caregiver, and (3) consequences of involvement in care for the personal life of the caregiver. Response options ranged from 1 (strongly agree) to 5 (strongly disagree), higher sum-scores indicate a higher sense of competence [33].

Mean changes across the follow-up time points were analyzed using descriptive statistics in SPSS [34]. Paired samples t-tests were conducted for both groups separately (high-active Inlife users vs. low-active users) to test the mean changes between baseline and 16-week follow-up (Table 6). Considering the explorative nature of this pilot study, small group sizes and missing values for some participants on several follow-up measurements, we could not perform reliable statistical tests to examine within- and between-subject effects.

**Table 6:** Results on primary outcome variables.

	Primary effects	Mean difference (SD):	Baseline—16-week Follow-up	
		Total (N = 23)	Low-active group (N = 17)	High-active group (N = 6)
**Perceived support (MSPSS)**				
	Perceived support friends	-.0.96 (3.51)	-1.12 (3.57)	-0.50 (3.62)
	Perceived support family	-2.26 (4.92)^a^	-3.18 (4.56)^a^	0.33 (5.39)
	Perceived support others	-0.83 (5.51)	-1.71 (5.87)	1.67 (3.62)
**Received support (SSL_12)**				
	Total received support	-1.39 (3.19) ^a^	-1.00 (3.48)	-2.50 (1.97)^a^
	Everyday support	-0.39 (1.62)	-0.47 (1.59)	-0.16 (1.83)
	Support problem situations	-0.43 (1.55)	-0.05 (1.60)	-0.33 (1.51)
	Esteem Support	-0.96 (1.61) ^a^	-0.59 (1.58)	-2.00 (1.26)^a^
**Loneliness (LS)**				
	Total loneliness	0.17 (2.15)	0.47 (2.31)	-0.67 (1.37)
	Emotional loneliness	-0.30 (1.82)	-0.06(1.85)	-1.00 (1.67)
	Social loneliness	0.48 (1.20)	0.53 (1.33)	0.33 (0.82)
**Sense of competence (SSCQ)**				
	Total sense of competence	0.52 (1.44)	0.47 (1.33)	0.67 (1.86)

^a^ P<0.05 using paired samples t-test between baseline and 16-week follow-up

**Results:** According to the paired samples t-test analyses (Table 6), the high-active Inlife users showed less of a decline after 16 weeks in perceived family support (mean difference = .33, Standard Error (SE) = 2.201, *P* = ns) than the low-active users (mean difference = -3.18, SE = 1.12, t_16_ = 2.9, *P* = .011). With regard to total received support, we found that the high-active Inlife user group had significantly lower levels of received support at 16-week follow-up (mean difference = -2.50, SE = .81, t_22_ = 3.1, *P* = .027) compared to the low-active Inlife user group (mean difference = -1.00, SE = .85, *P* = ns). However, due to the small sample size, we cannot exclude the possibility that these findings are due to other factors such as regression to the mean. The data indicated a trend towards improvements in feelings of competence and a decrease in feelings of loneliness (in the high-active Inlife users).

#### Adapting the final platform

The results of the feasibility study provided the basis for the further adaption of the Inlife platform. The Inlife website was re-designed into an independent application version for the tablet and smartphone to enable faster communication that became available alongside the existing web-based platform. Furthermore, by adding entries, the calendar was made to be more intuitive. Although the primary caregivers remained as the account administrator, their picture was repositioned from a central position above the dashboard to the inner circle to obtain more equality within the network circles. Short video clips with hands-on user instructions relating to each Inlife functionality were added.

## Discussion

The present paper describes the development and piloting process of Inlife, a web-based social support intervention for caregivers of individuals with dementia aiming to improve social support and caregiver feelings of competence. Consistent with former psychosocial intervention studies [25, 35], the MRC framework guided the development process, enabling the integration of existing research, theoretical frameworks, expert knowledge and users’ views. By including potential users in the development process, the Inlife platform was designed to fit the needs of caregivers and individuals with dementia.

Previous studies have highlighted the potential for Internet interventions to serve as a cost-effective alternative to caregiver support [36, 37]. Similarly, during the exploration of user views, caregivers reported positive attitudes towards online tools, as they might provide opportunities for anonymous interaction and information exchange with people at a distance. However, potential users were cautious due to privacy, security, and sometimes felt reluctant to access new, unfamiliar online programs. Therefore, during the development process, we ensured their privacy using personal passwords. Additionally, the platform was structured according to three circles with different privileges.

The results of our pilot study showed acceptable satisfaction rates based on questionnaire data and participant feedback. Overall, participants’ appreciation of Inlife was rated as good (M = 7.1, SD = 1.3). This rating differed between high-active (M = 8.0, SD = 0.7) and low-active users (M = 6.7, SD = 1.3). Most users were satisfied with the content and the number of functions on the Inlife platform. Participants reported that Inlife helped them to share their experiences and supported their coordination of the care process. Nevertheless, the rate of low-active users was high. This was partially due to the non-response of significant others in the Inlife networks, concurrent use of other online communication tools (e.g., WhatsApp), complex log-in procedures, and unfamiliarity with online tools. Adaptions were made to resolve these issues such as the development of an application version of Inlife for faster information exchange and the addition of instructional videos. Since both groups negatively evaluated the number of e-mails, adaptations were made so that caregivers could set personal preferences regarding the receipt of e-mail notifications. In general, both groups reported that the platform was not helpful for requesting support more easily. This finding reflects the prevailing stigma and threshold of seeking support [14, 15]. However, the shared notion that the platform helped caregivers to share more information in a more positive fashion (e.g., sharing pictures of daily activities) might be the first step towards gaining more openness and support.

Given the exploratory nature of this pilot study, we should be cautious when interpreting the preliminary results. We observed a promising trend towards improvements in perceived family support, sense of competence, and reduced feelings of loneliness in the high-active Inlife user group (Table 6). Surprisingly, received support declined more in the high-active Inlife user group. In this group, it might be possible that the Inlife intervention has triggered a heightened awareness of the lack of experienced social support. However, this might not be a robust finding due to the small sample size and underpowered tests. Therefore, this study should be replicated on a larger scale to include a control group. This enables evaluation of the overall positive and negative effects of Inlife and will reveal potential issues that need adaption to improve the final platform.

### Lessons learned and future directions

The high rate of low-active users of Inlife indicates that this web-based intervention might be more suitable for particular subgroups of caregivers. Compared to the high-active Inlife users, low-active users had significantly smaller network sizes. This finding is in line with previous studies that demonstrated that caregivers with a larger social network [38] and lower age [39] might benefit more from Internet interventions. Participants with a smaller Inlife network were less active, since they reported to receive less response from circle members on the Inlife platform. This is in line with research that demonstrated that support seeking is based on mutual reciprocity within interpersonal relationships and is influenced by both weak-tie (acquaintances, peers, neighbors) and strong-tie (friends, family) relationships [40]. Possibly, people with a small Inlife network have less interaction with weak-tie network members. Additionally, individuals with a small support network might perceive seeking support already as more burdensome, since barriers to accessing support are influenced by existing relational boundaries between friends and family and family dynamics [41]. Further research is needed to examine whether Inlife is beneficial for both caregivers with a small and a large social network.

Furthermore, the amount of time since diagnosis was found to be slightly shorter in the high-active users group. This finding is consistent with previous studies that showed that interventions at an early dementia stage might be more beneficial [16].

Our log data also demonstrated that some people continued to use the platform, whereas others stopped. Low-active Inlife users reported several reasons for their non-adherence such as the low motivation of other network members, low personal effort, and limited user guidance. Although non-adherence is not unusual in e-health interventions [42, 43], there might be some solutions to overcome this challenge. Our preliminary findings yielded valuable insights. For example, guidance by a professional moderator in settings that offer Inlife might be beneficial, as experts might be able to motivate care network members by sharing their knowledge and creating awareness of social support needs. Furthermore, guidance by a personal mediator or volunteer might provide practical hands-on advice regarding Inlife usage, thereby increasing treatment adherence and engagement. Previous studies already demonstrated beneficial effects of interaction with a personal coach [17]. The high rate of low-active Inlife users emphasizes the need for clear, step-by-step instructions not only for the primary coordinating caregiver, but also for people in the Inlife circles. Furthermore, future studies should provide more insight into the potential barriers and facilitators for the uptake of the Inlife intervention to allow for screening of caregivers who benefit most from the online program.

### Limitations

Selection bias was inevitable, as demonstrated by the relatively young age and high education level of the participants. This might be due to the online nature of the study. Furthermore, the sampling procedure might have limited the generalizability of our results. We excluded caregivers who were overburdened or experienced severe health problems to prevent additional burden caused by research participation. However, especially these caregivers might benefit from having access to online social support. Therefore, in future studies the inclusion criteria might be broadened. Potential users were closely involved in several but not in all parts of the development process. Ideally, researchers should consider including caregiver in all phases of the development process to ensure content validity. In the development phase, we focused on views of spousal caregivers to increase homogeneousness. It would have been valuable to include also the views of other network members with a more heterogeneous background. In accordance with the iterative development process the reports of both the participating adult-children and spouses were considered for adapting the final platform. There is no gold standard for the feasibility evaluation of web-based interventions. Therefore, aspects of the Inlife platform were evaluated by using descriptive mean item scores. In a larger scale study it would be valuable to apply qualitative interviews to gain in-depth insight in subjective user experiences, support satisfaction, and process characteristics. It was reported that the follow-up assessments interfered to some degree with the actual Inlife usage. Therefore, it could be considered to decrease the number of follow-up measurements. We assessed both the concepts of social support and feelings of loneliness, considering that socially supported persons might at the same time feel lonely or visa versa [44, 45]. However, both reports of social support and loneliness are subjective in nature. Therefore, in future studies more objective measures of social isolation (i.e., number of meaningful ties in one’s social network) could be assessed [46]. Although being outside the scope of the present study, it would also be interesting to measure the effects of Inlife on social support in individuals with dementia. However, it might be challenging to select a measure that reliably reflects their social support experiences and that is validated for individuals with dementia.

## Conclusions

The Inlife platform was shown to be feasible for informal caregivers of individuals with dementia and provided them with opportunities for positive interactions and support. Nevertheless, the uptake of the Inlife platform was not optimal. The results of this pilot study were used to improve the intervention. In line with the MRC framework, this feasibility study is the first step towards examining the effectiveness of the intervention in a randomised controlled trial. The Inlife platform is currently available (June 2016) to caregivers who are willing to participate in a follow-up effectiveness study.
